# Four cases of laparoscopic colectomy for sigmoid colon and rectal cancer with persistent descending mesocolon

**DOI:** 10.1186/s40792-020-00988-6

**Published:** 2020-10-02

**Authors:** Yumi Furuichi, Kensuke Kumamoto, Eisuke Asano, Akihiro Kondo, Jun Uemura, Hironobu Suto, Minoru Oshima, Takayoshi Kishino, Hisashi Usuki, Keiichi Okano, Yasuyuki Suzuki

**Affiliations:** Department of Gastroenterological Surgery, Faculty of Medicine, Kagawa University, 1750-1 Ikenobe, Miki-cho, Kita-gun, Kagawa, 761-0793 Japan

**Keywords:** Persistent descending mesocolon, Laparoscopic colectomy, Abnormal fixation

## Abstract

**Background:**

Persistent descending mesocolon (PDM) is a congenital anomaly associated with the failure of fixation of the descending colon to the lateral abdominal wall. In the laparoscopic colectomy for colorectal cancer, it has been noticed that there are extensive adhesions and a distinctive anatomy of colonic vessels in cases with PDM. Therefore, it is necessary to have sufficient knowledge about PDM so that it can be appropriately treated during surgery.

**Case presentation:**

Case 1—a 79-year-old man underwent laparoscopic intersphincteric resection for rectal cancer. Preoperative barium enema (BE) revealed that the sigmoid colon was located at the right side of the abdomen. An enhanced computed tomography (CT) showed that the common trunk of the left colic artery (LCA) and the first sigmoid colonic artery (S1) branched from the inferior mesenteric artery (IMA). Case 2—a 68-year-old man underwent laparoscopic sigmoidectomy for sigmoid colon cancer and laparoscopic distal gastrectomy for gastric cancer synchronously. BE showed that the descending colon ran from the splenic flexure to medial caudal side. An enhanced CT showed that the distance from the LCA to the marginal artery was 1.0 cm. Case 3—a 68-year-old man underwent laparoscopic low anterior resection for rectal cancer. BE showed that the descending colon ran to the medial caudal side. An enhanced CT showed that the mesentery of the descending colon was comparatively shortened. Case 4—a 60-year-old man underwent laparoscopic sigmoidectomy for sigmoid colon cancer. An enhanced CT showed that the descending colon ran to the medial caudal side and predicted that the LCA and S1 formed a common trunk and branched radially from the IMA. We reported four cases with PDM recognized preoperatively as above. Three cases had a shortening of the mesocolon. While dissecting the vessels, although special attention was required to maintain the blood flow to the intestine, none of these cases developed any complications during the postoperative course.

**Conclusions:**

We considered that it is important to have positional awareness of the LCA and the marginal artery to perform the laparoscopic surgery safely when a colorectal cancer with PDM is diagnosed preoperatively using imaging methods.

## Background

Persistent descending mesocolon (PDM), which was first reported by Morgenstern in 1960 [1], is defined as an anomaly of fixation of the mesentery of the descending colon, wherein the descending colon is located toward the medial side and the sigmoid colon is located at the right side of the abdomen. Morgenstern classified PDM into three types according to the degree of the displacement of descending colon and the range of the adhesion based on reported cases. Type A is the lack of fixation of both the ascending and descending colon along with the absence of the transverse colon, type B is moderate displacement of the descending colon to the midline or slightly left of the midline, and type C is the marked displacement of descending colon with paracecal fixation. In the era of laparoscopic colectomy for colorectal cancer, it has been noticed that there are extensive adhesions and a distinctive anatomy of colonic vessels in cases with PDM. Recently, several reports [2–8] have suggested tips and tricks for laparoscopic colectomy in colorectal cancer with PDM. We herein reported four colorectal cancer patients with PDM who underwent laparoscopic surgery along with the technical aspects, in terms of tips and tricks, including the characteristics of the preoperative images and the anatomical alterations of colorectal arteries and veins during the operation.

## Case presentation

### Case 1

**Fig. 1:**
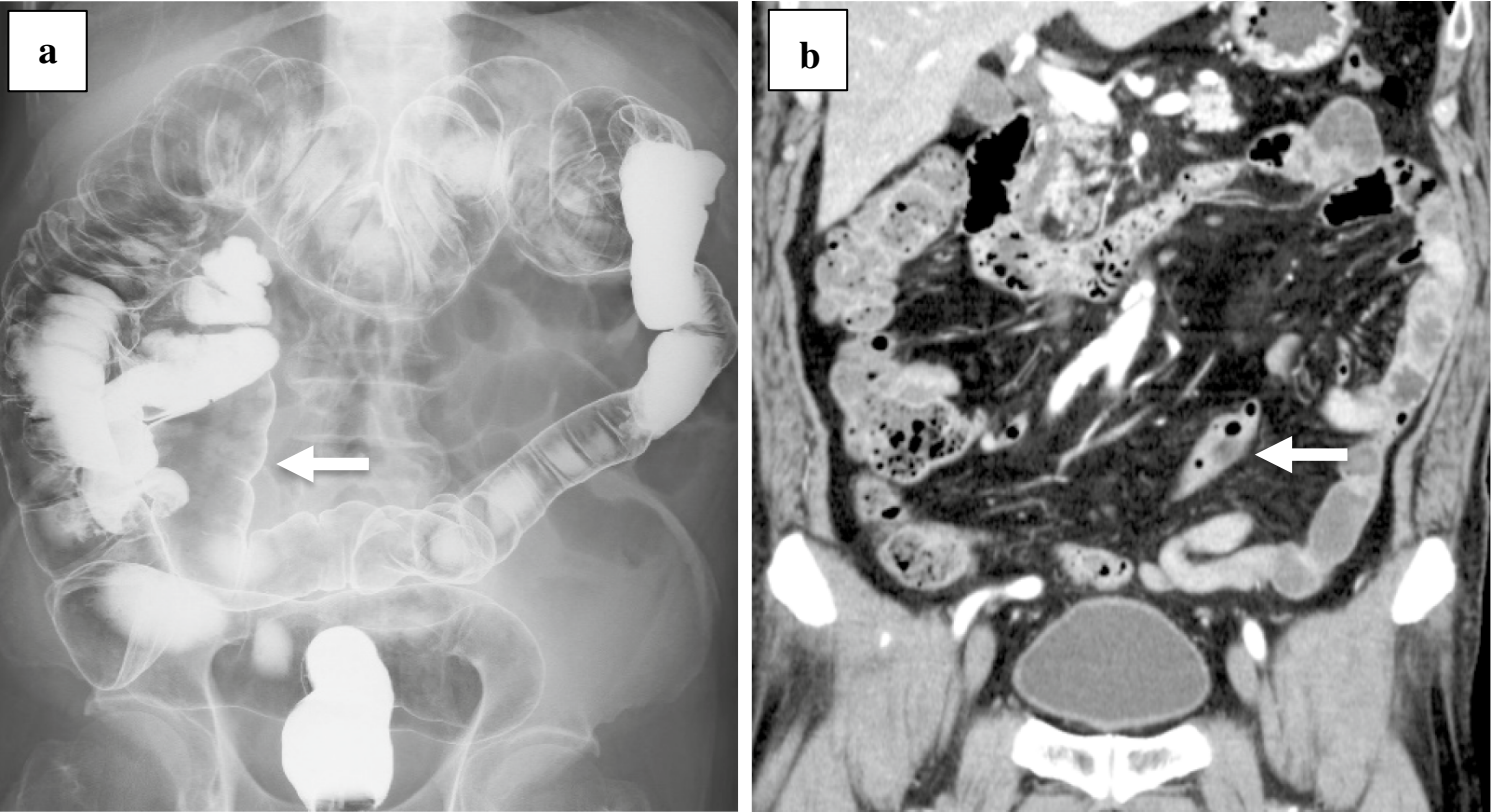
Case 1. **a** BE revealed the marked displacement of the descending colon with paracecal fixation (white arrow) and the sigmoid colon was on the right side of the abdomen. **b** An enhanced CT showed the descending colon (white arrow)

A 79-year-old man underwent laparoscopic intersphincteric resection for rectal cancer. The preoperative diagnosis was cT1N0M0 stage I (TNM classification, 8th edition). Preoperative barium enema (BE) revealed that the sigmoid colon was located at the right side of the abdomen (Fig. 1a). An enhanced computed tomography (CT) showed that the descending colon ran to medial caudal side (Fig. 1b), which indicated that it was type C based on the Morgenstern’s classification [1]. CT findings showed that the common trunk of the left colic artery (LCA) and the first sigmoid colonic artery (S1) branched from the inferior mesenteric artery (IMA). The operative findings showed that the sigmoid colon was long and adhered to the cecum, the ileum, and the mesentery of ileum to the jejunum. After adhesiolysis was performed to obtain a clear vision of the anatomy, the medial approach was performed. The common trunk of the root of LCA and S1, and superior rectal artery (SRA) branched from IMA radially; in this case, the SRA and S1 were resected and the LCA was preserved. No postoperative complications were observed.

### Case 2

**Fig. 2:**
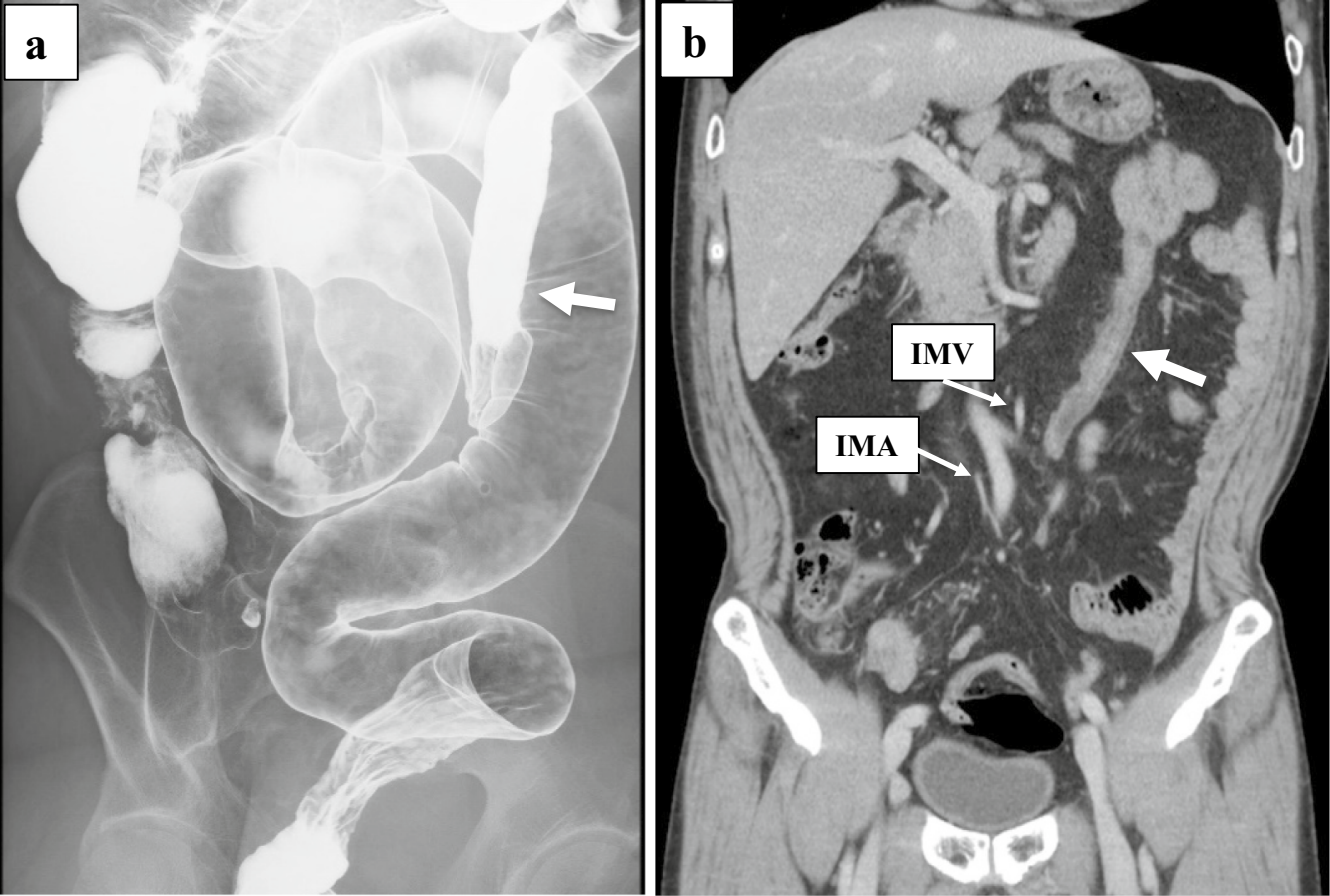
Case 2. **a** BE revealed moderate displacement of the descending colon to the midline (white arrow) and the sigmoid colon was quite long. **b** In an enhanced CT, the white arrow showed the descending colon. IMA, IMV, and the descending colon ran very close to each other

A 68-year-old man underwent laparoscopic sigmoidectomy for sigmoid colon cancer and laparoscopic distal gastrectomy for gastric cancer synchronously. The preoperative diagnosis of sigmoid colon cancer was cT3N0M0 stage IIA (TNM classification, 8th edition). The BE findings indicated that the descending colon ran from the splenic flexure to medial caudal side (Fig. 2a). This case was categorized as type B of the Morgenstern’s classification. An enhanced CT showed IMA, IMV, and the descending colon ran very close to each other. The operative findings showed that the mesentery of the descending colon was shortened and the left ureter was recognized on the lateral side of the descending colon. The sigmoid colon widely adhered to the mesentery of the small intestine. After the resection of the IMA, the rest of the procedure was performed under direct vision. The LCA, S1, and SRA branched radially from the IMA. The LCA and IMV were resected at the same level of IMA resection while confirming the run of the marginal artery. The patient was discharged without any complications.

### Case 3

**Fig. 3:**
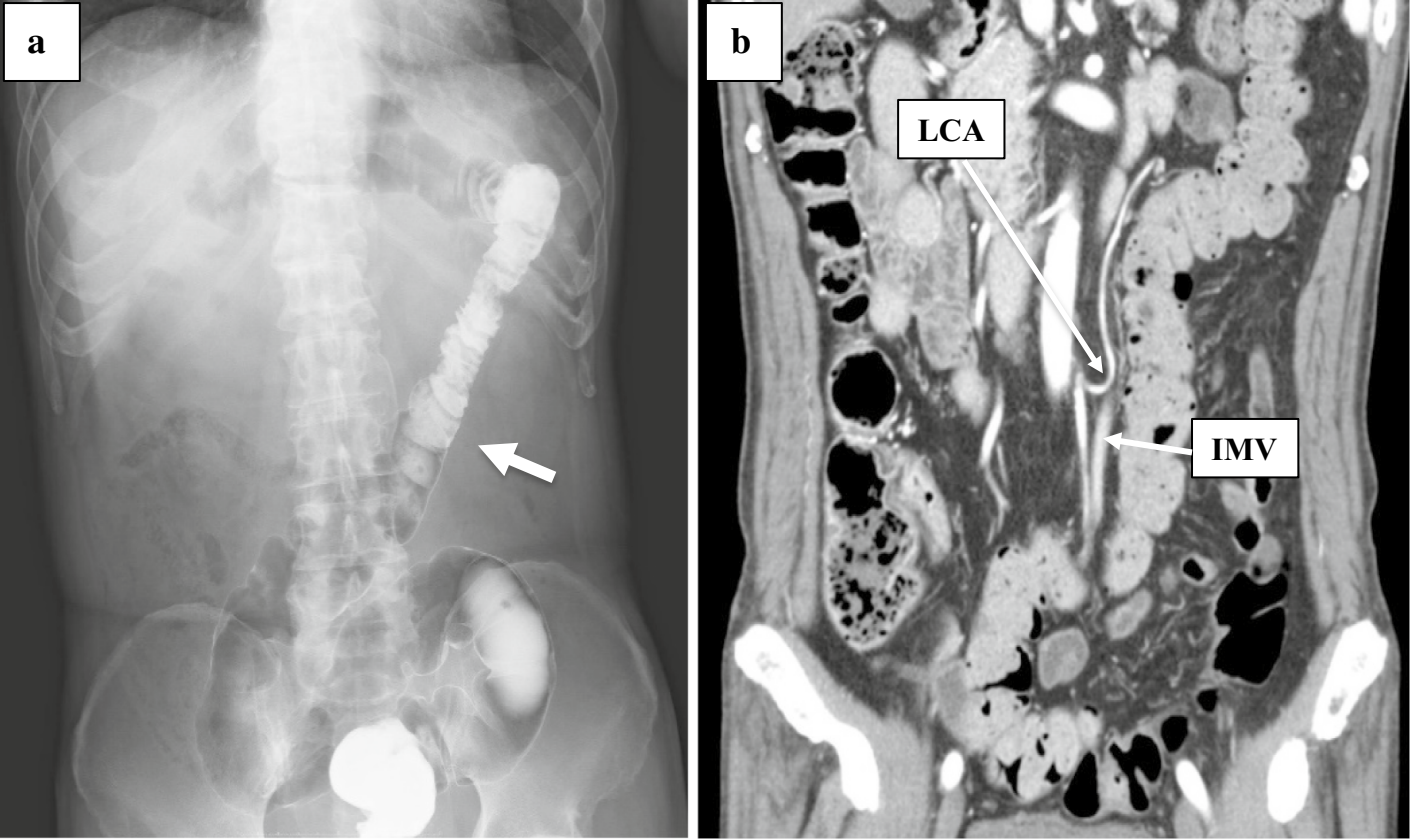
Case 3. **a** BE revealed moderate displacement of the descending colon to the midline (white arrow). **b** An enhanced CT showed shortening of the descending mesocolon. LCA, IMV, and marginal vessels ran very close to each other

A 68-year-old man underwent laparoscopic low anterior resection for rectal cancer. Preoperative diagnosis was cT3N1aM0 stage IIIB (TNM classification, 8th edition). Preoperative BE imaging showed that the descending colon ran to the medial caudal side, which identified it as type B of the Morgenstern’s classification (Fig. 3a). An enhanced CT showed that the mesentery of the descending colon was comparatively shortened and the length from the LCA to the marginal artery (with the IMV located behind them) was about 3–9 mm (Fig. 3b). As observed in the preoperative images, the shortening in the mesentery of the descending colon and the adhesion of the sigmoid colon to the mesentery of the small intestine were recognized in the operative findings. After adhesiolysis was performed and mobilization of the sigmoid colon was obtained, the medial approach of the sigmoid colon was performed as usual. The IMA was ligated during the laparoscopic procedure, which was followed by ligation of the LCA and IMV under direct vision. The branch of S1 was more peripheral side than that of LCA. No complications were observed in postoperative course.

### Case 4

**Fig. 4:**
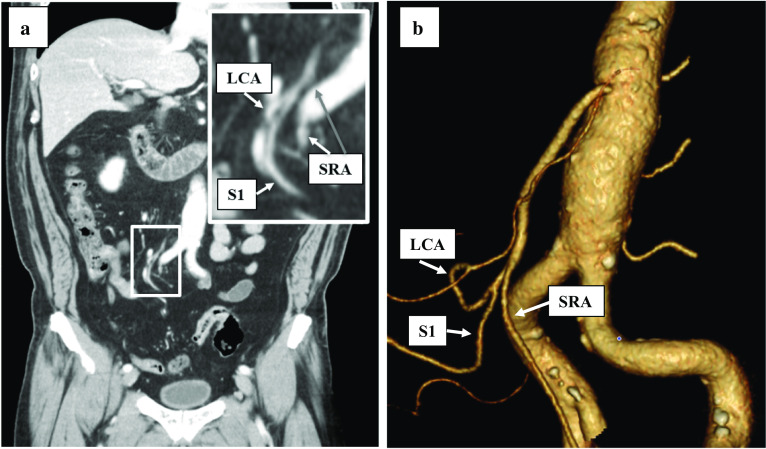
Case 4. **a** An enhanced CT showed LCA and S1, SRA branched radially from IMA. Enlarged view is shown. **b** 3D-CT showed LCA and S1, SRA branched radially from IMA

A 60-year-old man underwent laparoscopic sigmoidectomy for sigmoid colon cancer after endoscopic mucosal resection. His preoperative diagnosis was cT1N0M0 stage I (TNM classification, 8th edition). An enhanced CT showed that the descending colon ran to the medial caudal side and that the long sigmoid colon was placed at the right side of the pelvic cavity, which indicated that it was type B based on the Morgenstern’s classification. CT findings predicted that the LCA and S1 formed a common trunk and branched radially from the IMA (Fig. 4a, b). Considering the operative findings, the root of IMA was not observed visually during laparoscopic procedure because of the extensive adhesion of the descending and sigmoid colon to the small intestine. An adhesiolysis was first performed to find the root of the IMA. The LCA, S1, and SRA branched radially from the IMA. The common trunk of LCA and S1 was preserved and the root of the SRA was resected. The patient was discharged without any postoperative complications.

## Discussion

We successfully treated four cases of colorectal cancer with PDM laparoscopically without any postoperative complication. To enable safe laparoscopic surgery, we focused on accurate preoperative diagnosis of PDM and presented the appropriate information regarding the anatomical features of PDM in this case report, through the review of previous literature. We searched PubMed using the following key words: “persistent descending mesocolon” and “cancer”. Consequently, seven reports were selected from those published between 1960 and 2020. All the reports were from Japanese institutions within a single decade (Table 1).

**Table 1:** Reported cases of colorectal cancer with PDM

Reported author	Reported year	Case number	Age (years)	Gender	Tumor location	Preoperative imaging methods	Preoperative diagnosis	Morgenstern's classification	Operation method	Operating time	Bleeding volume	Postoperative complications	LCA retention
Okada [2]	2013	13	67^a^	M10, F3	S9, R4	nd	nd	nd	Laparoscopic	239^a^	50^a^	1/13 (1; surgical site infection)	nd
Tsuruta [3]	2014	1	60	M	A	CT	**+ **	A	Laparoscopic	nd	nd	No	nd
Arai [4]	2016	1	65	M	S	CT-colonography	**+ **	B	Laparoscopic	272	49	No	**+ **
Mori [5]	2018	1	88	F	D	CT	**+ **	B	Laparoscopic	nd	nd	No	**━**
Hisano [6]	2019	1	80	M	S, R	CT, BE, CT-colonography	**+ **	A	Laparoscopic	nd	nd	No	**━**
Hiyoshi [7]	2019	2	50	M	S	CT-colonography	**+ **	C	Laparoscopic	835	285	Anastomotic stenosis	**━**
			77	M	R	CT-colonography	**+ **	C	Laparoscopic	333	10	No	**+ **
Wang (8)	2020	60	67^a^	M39, F21	nd	CT	5/60 (8.9%)	Unknown	Laparoscopic	Unknown	Unknown	11/60 (5; anastomotic leakage)	36/60 (36%)
Our cases	2020	4	79	M	R	CT, BE	**+ **	C	Laparoscopic	554	178	No	**+ **
			68	M	S	CT, BE	**+ **	B	Laparoscopic	237	0	No	**━**
			68	M	R	CT, BE	**+ **	B	Laparoscopic	386	56	No	**━**
			60	M	S	CT	**+ **	B	Laparoscopic	312	30	No	**+ **

^a^Mean

Generally, radiological imaging studies including enhanced CT and BE are routinely performed to obtain supportive information of colorectal cancer prior to surgical treatment. In our institution, all the four reported cases underwent an enhanced CT, which result in the diagnosis of PDM. Regarding CT reconstruction, coronal planes were useful to detect the displacement of the descending colon and the right-sided sigmoid colon. Besides the enhanced CT examination, BE seemed to better visualize the run of the bowel. Recently, CT-colonography has become a popular diagnostic imaging method than BE and colonoscopy [9]. As shown in Table 1, three reports [4, 6, 7] showed the use of a CT-colonography to detect colorectal cancer with PDM; the three-dimensional (3D) images helped to make a diagnosis of PDM. Enhanced CT was routinely performed in other cases for supportive clinical information. Wang et al. [8] reported that preoperative detection was achieved in 5 (8.9%) out of 60 colorectal cancer patients with PDM. Therefore, even when PDM is not diagnosed by preoperative examinations in colorectal cancer patients, it is necessary to have sufficient knowledge about PDM so that it can be appropriately treated during surgery.

PDM is defined as a developmental anomaly that results from the failure of the descending mesocolon to fuse with the posterior parietal peritoneum by the end of the fifth month of gestation [1]. It has been reported that the frequency of PDM was 2.4% in patients who underwent laparoscopic colectomy for left-sided colorectal cancer [2]. Moreover, that was 2.1% in patients who underwent laparoscopic colectomy for primary colorectal cancer [8]. In our institution, the frequency of PDM was 1.9% in 209 patients who had laparoscopic colectomy for left-sided colorectal cancer from January 2014 to December 2019, while that was 1.3% in 307 all the colorectal cancer patients receiving laparoscopic surgery during same period. Although the difference in frequency might be influenced by regional characteristics, PDM was found at a similar rate in left-sided colorectal cancer patients.

Based on the Morgenstern’s classification, case 1 corresponded with type C and the remaining cases fit the definition of type B. As shown in Table 1, previous reports included two cases of each type [3–7]. Besides the Morgenstern classification, Okada et al. [2] categorized PDM into long-S type and short-S type according to the length of the sigmoid colon and adhesion to the descending colon. Long-S type has excessive adhesion between long sigmoid colon and descending colon. In the short-S type, descending colon runs straight without any adhesion with sigmoid colon. In the view of complexity of surgical procedure of adhesiolysis, this classification is also useful information for surgical treatment, especially in the case of laparoscopic surgery. The causes of excessive adhesion in patients with long-S type PDM have been still unknown. Adhesion usually occurs after abdominal surgery. In that case, it is known to occur in the process of repair by damage to the serosa of the intestinal tract. However, the adhesion of PDM is congenital anomaly of fixation without past history of laparotomy. A previous report [10] described that the adhesion of the mesentery of colon was found in a 3-month-old boy with PDM. The adhesion might have occurred at the early stage in fetus. Another report [11] indicated that frequent midgut volvulus caused the adhesion. The patients with PDM may have undergone volvulus of left-sided colon to the right-sided mesentery of the intestine several times and repeated spontaneous reductions, possibly resulting in adhesions to the mesentery of the small intestine and/or cecum.

With increasing morbidity of colorectal cancer in recent years, laparoscopic surgery has become popular worldwide. In laparoscopic colorectal surgery in the case of PDM with a wide range of adhesion, it is necessary to perform adhesiolysis first to make the anatomy of the colon clear. All the four presented cases were classified as the long-S type, where the sigmoid colon adhered to both the descending colon and the right side of the pelvic cavity. Although it takes time to dissect this adhesion, unlike the inflammatory adhesion, it is possible to complete it through laparoscopic procedure because the division line is usually visualized. Additionally, PDM has no Toldt’s fusion fascia because of the failure of the descending mesocolon to fuse with the retroperitoneum, which leads to the formation of a shortened mesocolon. Hence, the left ureter and the gonadal vessels can be seen through the retroperitoneum. The most important considerations for the surgical treatment of colorectal cancer with PDM are the positional awareness of vessels including the IMA, LCA, and marginal artery in the shortened mesocolon. In PDM cases, the configuration of IMA branching were reported that LCA, S1 or second sigmoid artery (S2), and superior rectal artery (SRA) branched radially from IMA, referred to as “bear-claw IMA” [8]. Wang et al. [8] reported that LCA was usually much shorter and may configure directly as a part of the marginal artery under those branching patterns. In two of the four cases (cases 2 and 3), LCA was close to the marginal artery in the preoperative and operative findings and the procedure of LCA ligation was performed under direct vision after the resection of IMA laparoscopically. Although the laparoscopic enlarged view would be better than direct vision to resect LCA and IMV, we performed it under direct vision because it was difficult to identify the blood vessels laparoscopically. Arai et al. also recommended that the vessel resection, except for IMA, should be performed under direct vision in a PDM case [4]. Certainly, preserving LCA could be beneficial to maintain the intestinal blood flow so that anastomotic reconstruction can be performed safely. The reconstruction was performed using double-stapling technique in all of the four cases. Recently, Hiyoshi et al. [7] reported that performing intraoperative infrared ray imaging using indocyanine green was useful for confirmation of the blood flow to the reconstructed oral side of the colon prior to the anastomosis. The IR imaging method is considered suitable for the intraoperative evaluation of blood flow in colorectal cancer cases with PDM.

## Conclusions

PDM with colorectal cancer can be detected preoperatively using anatomical information from imaging devices such as an enhanced CT, BE, and CT-colonography. Furthermore, the positional awareness of vessels related to IMA is essential before performing a laparoscopic surgery. When LCA resection is required for proper lymph node dissection in advanced colorectal cancer, surgeons have to pay special attention to the resection of LCA to prevent anastomotic complications that can occur because of an impairment of blood flow.

## Data Availability

The authors declare that all the data in this article are available within the article.

## Abbreviations

PDM: Persistent descending mesocolon
BE: Barium enema
CT: Computed tomography
LCA: Left colic artery
S1: First sigmoid colonic artery
S2: Second sigmoid artery
IMA: Inferior mesenteric artery
IMV: Inferior mesenteric vein
SRA: Superior rectal artery
